# Cyclohexene oxide CA, a derivative of zeylenone, exhibits anti-cancer activity in glioblastoma by inducing G0/G1 phase arrest through interference with EZH2

**DOI:** 10.3389/fphar.2023.1326245

**Published:** 2024-01-09

**Authors:** Rui Su, Weiwei Cao, Guoxu Ma, Weiping Li, Zongyang Li, Yongpei Liu, Lei Chen, Zebin Chen, Xuejuan Li, Ping Cui, Guodong Huang

**Affiliations:** ^1^ Department of Neurosurgery, Shenzhen Second People’s Hospital/The First Affiliated Hospital of Shenzhen University Health Science Center, Shenzhen, China; ^2^ Institute of Pharmacy, Shenzhen University Medical School, Shenzhen, China; ^3^ Institute of Medicinal Plant Development, Peking Union Medical College and Chinese Academy of Medical Sciences, Beijing, China; ^4^ Department of Pharmacy, Shenzhen Children’s Hospital, Shenzhen, China

**Keywords:** glioblastoma, zeylenone, enhancer of zeste homolog 2, G0/G1 arrest, polycomb repressive complex 2

## Abstract

**Introduction:** Due to its highly aggressiveness and malignancy, glioblastoma (GBM) urgently requires a safe and effective treatment strategy. Zeylenone, a natural polyoxygenated cyclohexenes compound isolated from *Uvaria grandiflora*, has exhibited potential biological activities in various human diseases, including tumors.

**Methods:** We designed and synthesized a series of (+)-Zeylenone analogues and evaluated their anti-GBM roles through structural-activity analysis. Cell Counting Kit-8, TUNEL, transwell and flow cytometry were employed for investigating the anticancer effects of CA on GBM cells. Western blotting, molecular docking, qRT-PCR and ChIP assays were performed to reveal the underlying mechanisms by which CA regulates the GBM cell cycle. The nude mouse xenograft model, HE staining, immunohistochemistry and was used to evaluate the anticancer effect of CA *in vivo*.

**Results:** We identified CA ((1R, 2R, 3S)-3-p-fluorobenzoyl-zeylenone) as having the lowest IC_50_ value in GBM cells. CA treatment significantly inhibited the malignant behaviors of GBM cells and induced G0/G1 phase arrest *in vitro*. Furthermore, we validated the molecular mechanism by which CA interferes with EZH2, attenuating the down-regulation of cyclin-dependent kinase inhibitors p27 and p16 by the PRC2 complex. By establishing orthotopic nude mice models, we further validated the inhibitory role of CA on tumorigenesis of GBM cells *in vivo* and its potential values to synergistically potentiate the anti-tumor effects of EZH2 inhibitors.

**Conclusion:** Overall, this paper elucidated the anti-GBM effects and potential mechanisms of CA, and may provide a therapeutic drug candidate for GBM treatment.

## Introduction

Gliomas are the most common form of central nervous system (CNS) cancers in adolescents, accounting for approximately 70% of primary intracranial tumors, and are classified as grades I–IV according to the World Health Organization (WHO) classification. Glioblastomas (GBMs), classified as WHO grade IV, represent the most malignant gliomas, with a median survival time of within 16 months (Chen et al., 2021). Despite significant advances in treatment strategies, the prognosis for GBM remains bleak, and this is primarily attributed to its highly aggressive, vascularized, and infiltrating pathological characteristics. In recent years, there has been a growing recognition of the involvement of epigenetic regulation in the pathological processes of GBMs. Several associated regulators have been identified as promising drug targets, offering potential avenues for therapeutic intervention (Ciftci et al., 2021; Wang et al., 2021).

Previous studies have elucidated that enhancer of zeste homolog 2 (EZH2), a histone lysine methyltransferase, plays complex roles in tumorigenesis and cancer development by influencing various cellular processes, including cell cycle progression, autophagy, and apoptosis (Duan et al., 2020). As a key subunit of polycomb repressive complex 2 (PRC2), EZH2 can alter chromatin compaction and the expression level of specific genes by methylating histone H3 lysine 27 (H3K27me3) (Laugesen et al., 2019). Consequently, numerous EZH2 inhibitors have been developed and recognized as effective anti-tumor agents in cancers. For instance, 3-deazaneplanocin A (DZNep) robustly impedes the self-renewal and tumor-forming capabilities of GBM stem cells by inducing pharmacological disruption of EZH2 (Suva et al., 2009). In a single-arm clinical study, tazemetostat, an oral EZH2 inhibitor, demonstrated significant therapeutic efficacy and safety in follicular lymphoma treatment, particularly in the EZH2 wild-type cohort (Morschhauser et al., 2020). Another novel molecule, *n*-butylidenephthalide, has been validated for its ability to inhibit the tumorigenic potential in GBM stem cells by decreasing AXL/EZH2 (Yen et al., 2017).

Naturally occurring products have been regarded as the most abundant source for developing clinical drugs due to their high biological activity and diversity. The results claimed that up to 70% of anti-cancer drugs are synthesized from natural products or derived from plants (Agarwal et al., 2020; Sun et al., 2021). Zeylenone is a naturally occurring cyclohexene oxide that was first isolated from the extract of *Uvaria grandiflora* leaves (Yang et al., 2018). Notably, zeylenone has demonstrated robust anti-tumor activities against cancer cells in cervical carcinoma, gastric cancer, and prostate cancer, with limited cytotoxicity toward normal cell lines (Zhang et al., 2017; Zeng et al., 2018). However, *in vivo* pharmacokinetic studies have revealed that zeylenone is susceptible to esterase hydrolysis, leading to the formation of benzoic acid metabolites that lose their potential biological activity. Furthermore, the potential roles of zeylenone in GBM remain unclear.

In the present study, we designed and synthesized a series of (+)-zeylenone analogs, ultimately pinpointing compound CA ((1R, 2R, 3S)-3-p-fluorobenzoyl-zeylenone) as the most potent anti-tumor agent against GBM cells. Our findings revealed that CA significantly increased the expression of cyclin-dependent kinase inhibitors (CDKIs) p27 and p16, leading to the arrest of GBM cells in the G0/G1 phase of the cell cycle. This effect was attributed to CA’s ability to diminish the inhibitory role of PRC2 through disruption of EZH2 participation. Our study identified CA and validated its potential value in GBM treatment both *in vitro* and *in vivo*, which would lead to new strategies and open new avenues for GBM treatment.

## Materials and methods

### Synthesis of zeylenone and its derivatives

Zeylenone was first protected by 2,2-dimethoxypropane and then dissolved in 5 mL DCM. Triethylamine (0.6 mL, 4 mmol), DMAP (25 mg, 0.2 mmol), and benzoyl chloride (360 μL, 3 mmol) were added successively to the mixture. After stirring for 2 h at room temperature, the reaction was quenched with saturated sodium bicarbonate solution, and the resulting mixture was extracted with DCM, washed with water, dried (Na_2_SO_4_), and concentrated under reduced pressure. The crude product was purified by silica gel column chromatography to obtain different derivatives. The synthesis and characterization details of (+)-zeylenone analogs including CA are described in Supplementary Material S1.

### Cell culture and the generation of patient-derived GBM strains

Human malignant GBM cell lines (U251, U87, A173, and U343) and human astrocyte (HA) cells were obtained from the Institute of Biochemistry and Cell Biology (Shanghai, China) and maintained in Dulbecco’s modified Eagle medium (DMEM, Life Technologies/Gibco, United States) containing 10% fetal bovine serum (FBS, Life Technologies/Gibco, United States).

The primary GBM-20-07 and GBM-20-13 cells were derived from the surgical specimens of patients diagnosed as WHO grade IV by the Department of Neurosurgery, The First Affiliated Hospital of Shenzhen University. The Ethics Committee of The First Affiliated Hospital of Shenzhen University approved the study, and informed consent was obtained from patients. The fresh tissues were washed using phosphate-buffered saline (PBS), cut into pieces using eye scissors, and digested with trypsin without ethylenediamine tetraacetic acid (EDTA). After being placed in the cell incubator for 15 min, the digests were removed, homogenized, and filtered using a 50-μm filter and then resuspended with DMEM/F-12 with 10% FBS. All cells were cultured in a humidified incubator containing 5% CO_2_ at 37°C.

### Cell transfection

In order to generate EZH2-knockdown cells, U251 cells were seeded in 24-well plates and transfected with sh-EZH2 plasmids (GenePharma, China) using Lipofectamine 3000 (Invitrogen, United States), according to the manufacturer’s instructions. Stable transfected cells were selected in 5 μg/mL puromycin dihydrochloride (Sigma-Aldrich, United States). The transfection efficiency was then evaluated using quantitative real-time PCR (qRT-PCR) and Western blot analysis. The details of the shRNA sequences are listed in Supplementary Material S2.

### Cell viability assay

The cells (1,000/well) were seeded in 96-well plates and allowed to adhere for 24 h before the treatment. Subsequently, the cells were treated with escalating concentrations of CA or DMSO, and cell viability was assessed 48 h post-treatment using the cell counting kit-8 (DOJINDO, Japan). To evaluate apoptotic changes, the presence of mono- and oligonucleosomes in the cytoplasm of primary GBM cells was evaluated using the Cell Death Detection ELISA Kit (Roche, United States). According to the manufacturer’s protocol, the cells were collected in 400 µL of buffer and incubated with lysates and horseradish peroxidase-conjugated DNA antibodies for 72 h. Finally, the absorbance at 405 nm was measured using a microplate reader.

### Cell cycle analysis

The cells were seeded and grown in 60-mm culture plates and treated with increasing concentrations of CA or DMSO for 48 h, respectively. The cells were then trypsinized, collected, and washed using phosphate-buffered saline (FBS). After resuspending the cells, pre-cooled absolute ethanol was added to fix cells overnight. According to the manufacturer’s instruction of Cell Cycle and Apoptosis Analysis Kit (Beyotime, Jiangsu, China), 0.5 mL staining buffer, 25 µL propidium iodide staining solution (20X), and 10 µL RNase A (50X) were added to each sample, and the cell cycle distribution (5,000 events) was analyzed using flow cytometry (Beckman Coulter, United States) after incubation for 30 min.

### Western blot analysis

Cell lysates were harvested, washed, and lysed using RIPA buffer and then centrifuged at 13,000 rpm for 45 min at 4°C (Vernet et al., 2023). The protein concentration was determined using the BCA Protein Assay Kit and diluted in loading buffer (4X). Equal amounts of the protein samples were subjected to SDS-PAGE and electrophoretically transferred onto PVDF membranes. Membranes were then blocked in Tween–Tris-buffered saline containing 5% (w/v) non-fat milk for 2 h at room temperature and incubated with the primary antibodies at 4°C overnight. The primary antibodies used were as follows: EZH2 (1:1,000, 21800-1-AP, Proteintech, United States), p16 (1:800, 10833-1-AP, Proteintech, United States), p27 (1:1,000, 25614-1-AP, Proteintech, United States), SUZ12 (1:1,000, 20366-1-AP, Proteintech, United States), H3K27me3 (1:500, ab6002, Abcam, United States), and GAPDH (1:5,000, 10494-1-AP, Proteintech, United States). Membranes were then washed and incubated with secondary antibodies. The blots were finally visualized using SuperSignal™ West Pico PLUS (Thermo Fisher Scientific, United States) and scanned using ChemiScope Capture software. The relative integrated density values (IDVs) were calculated using ImageJ software.

### Migration and invasion assays

Transwell assays were performed to evaluate the migration and invasion abilities of cells. In brief, 2 × 10^5^ cells were resuspended in serum-free DMEM and seeded into upper transwell chambers (8 μm, pre-coated with or without Matrigel) and incubated upon lower chambers with 500 μL of 10% FBS medium at 37°C for 48 h. Finally, the cells that migrated or invaded were fixed with methanol and glacial acetic acid at a ratio of 3:1, stained with 20% Giemsa, and examined.

### Molecular docking

A molecular docking study was performed to investigate the binding mode between CA and the human EZH2 using AutoDock Vina 1.1.2 (Trott et al., 2010). The three-dimensional (3D) structure of EZH2 (PDB ID: 6C23) was downloaded from the RCSB Protein Data Bank (www.rcsb.org). The 2D structure of CA was drawn using ChemBioDraw Ultra 14.0 and converted to 3D structure using ChemBio3D Ultra 14.0 software. The AutoDockTools 1.5.6 package (Sanner, 1999; Morris et al., 2009) was employed to generate the docking input files. The ligand for docking was prepared by merging non-polar hydrogen atoms and defining rotatable bonds. The search grid of the EZH2 site was identified as center_x: 147.366, center_y: 105.771, and center_z: 141.307 with dimensions size_x: 47.25, size_y: 47.25, and size_z: 47.25, respectively. In order to increase the docking accuracy, the value of exhaustiveness was set to 16. For Vina docking, the default parameters were used if it was not mentioned. The best scoring pose as judged by the Vina docking score was chosen and visually analyzed using PyMOL 1.7.6 software (www.pymol.org).

### TUNEL assay

A terminal deoxynucleotidyl transferase-mediated biotinylated UTP nick end-labeling (TUNEL) reaction was carried out, according to the manufacturer’s instruction. In brief, the cells were treated with DMSO or CA (5/6/7 μM) for 24 h, followed by fixation with 4% paraformaldehyde for 30 min and treatment with 0.3% Triton X-100 for 30 min at room temperature. According to the TUNEL Apoptosis Assay Kit (BeyoClickTM, China), the cells were co-incubated with a click reaction solution for 60 min at room temperature in a dark environment, then scanned randomly, and visualized using an inverted fluorescence microscope (ZEISS LSM 900 Confocal Laser Scanning Microcopy, Germany).

### RNA extraction and quantitative real-time PCR

In brief, approximately 7*10^−5^ to 8*10^−5^ cells that were cultured in 35-mm six-well plates were collected, and the total RNA was extracted using the TRIzol reagent (Sigma, United States) and quantified by detecting the 260/280 nm ratio of RNA using a NanoDrop spectrophotometer. qRT-PCR was carried out using One-Step SYBR PrimeScript RT-PCR Kit (Takara Bio), following the manufacturer’s protocol. Experimental details and primers used are shown in Supplementary Material S2.

### Chromatin immunoprecipitation assay

The chromatin immunoprecipitation (ChIP) assay was performed using the SimpleChIP Enzymatic Chromatin IP Kit (Cell Signaling technology, United States), following the manufacturer’s protocol. In brief, approximately 1*10^−7^ cells were cross-linked, quenched, and collected in lysis buffer. Then, the lysates were incubated with rabbit IgG, EZH2, and H3 (trimethyl K27) antibodies (Abcam, United States) with rotation. Immunoprecipitated DNA was quantified using RT-PCR (the primer sequences for *CDKN1B*/p27 and *CDKN2A*/p16 are provided in Supplementary Material S2) and verified using agarose gel electrophoresis.

### Animal studies

All animal procedures were conducted in accordance with Guidelines for Experimental Animals of Shenzhen University and were approved by the Ethics Committee of Shenzhen University Health Science Center (No. 20201105001-FS01). Four-week-old female BALB/c nude mice were purchased from Beijing Vital River Laboratory Animal Technology Co., Ltd. For intracranial orthotopic inoculation, mice were anesthetized by administering 1% sodium pentobarbital (70–100 mg/1 kg body weight) intraperitoneally, fixed to a stereotaxic apparatus, and shaved. All mice were randomized into four groups (n = 5 each group). Subsequently, 5*10^5^ U251 cells, which were pre-transfected with plasmids and luciferase lentivirus, were injected into the right caudate nucleus of each subject. Mice in control and CA groups were injected with sh-NC-transfected cells, while the other two groups were injected with EZH2-knockdown cells. Five days after the injection, mice in the control and sh-EZH2 + DMSO groups received 50 μL DMSO intraperitoneally every 2 days. Mice in the sh-NC + CA and sh-EZH2 + CA groups received 50 μL of CA (10 mg/kg/2 days) intraperitoneally. Bioluminescence images of mice were recorded every 7 days after the treatment, and survival curves were generated. The treatment was continued for 35 days until all mice were euthanized, and all tumors were removed for immunofluorescence and H&E staining.

### Statistical analysis

Each experiment was performed independently with at least three replicates. All data are presented as the mean ± standard error of the mean (SEM). Two-tailed Student’s t-test and one-way ANOVA were performed to determine statistical differences using GraphPad Prism 8 and SPSS 17.0 software. *p* < 0.05 was considered statistically significant. The results from correlation analysis and Kaplan–Meier survival curves were interpreted using GraphPad Prism 8.

## Results

### The anti-tumor activities of designed (+)-zeylenone derivatives in GBM

Zeylenone, a naturally occurring anti-tumor agent, is a typical polyoxygenated cyclohexene compound isolated from *U. grandiflora* Roxb. Both (−)-zeylenone and its enantiomer (+)-zeylenone have demonstrated anti-tumor activities in various tumor cells, where the hydroxyls at the C-1/2 position and the *α,β*-unsaturated ketone at the C-5, 6 position play a crucial role. Additionally, the benzoyl group at the C-1 position inhibits the further hydrolysis of molecules, improving the action time of drugs (Sun et al., 2021).

**FIGURE 1:**
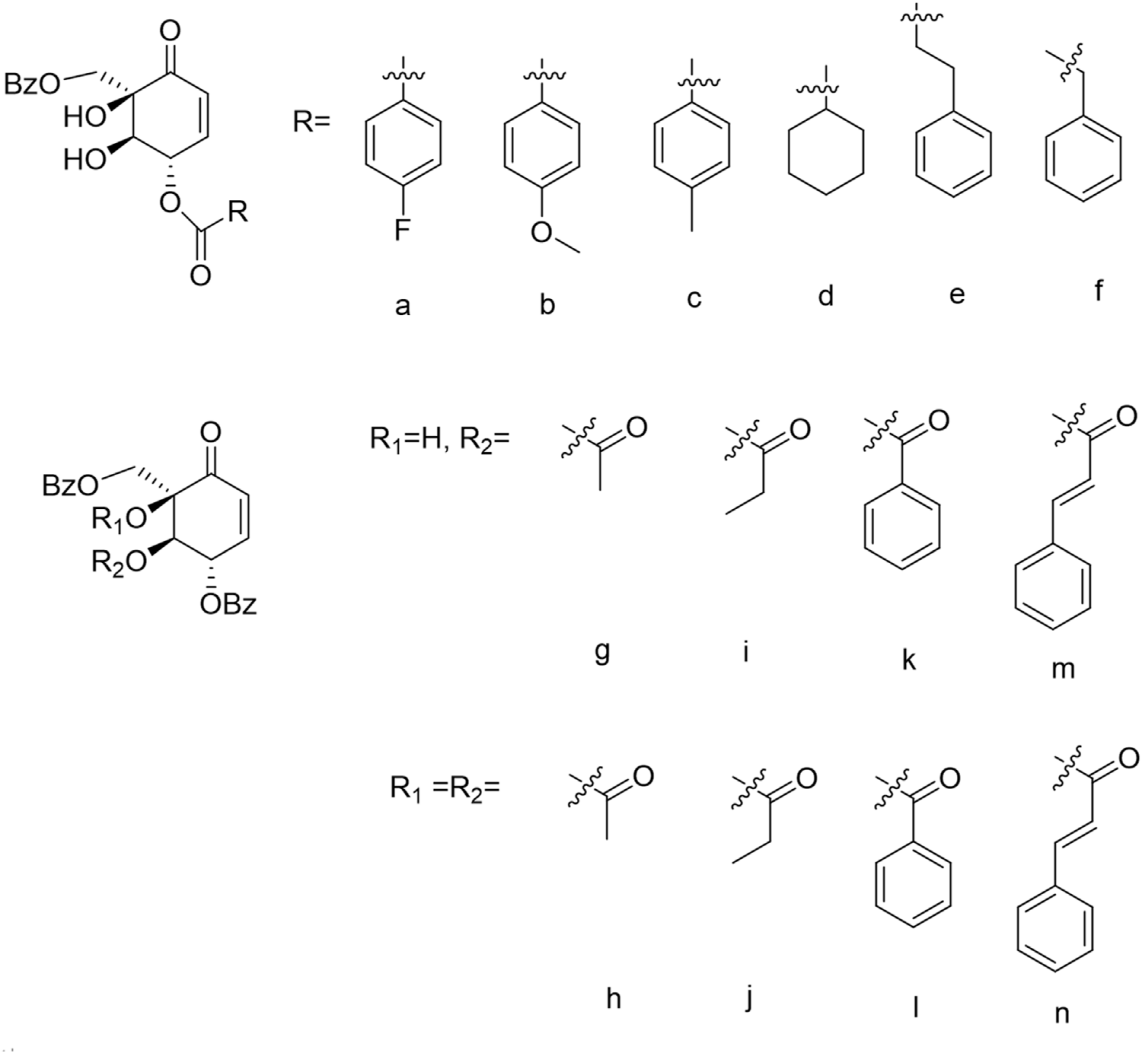
Synthetic design of analogs of (+)-zeylenone **(A-N)**.

**FIGURE 2:**
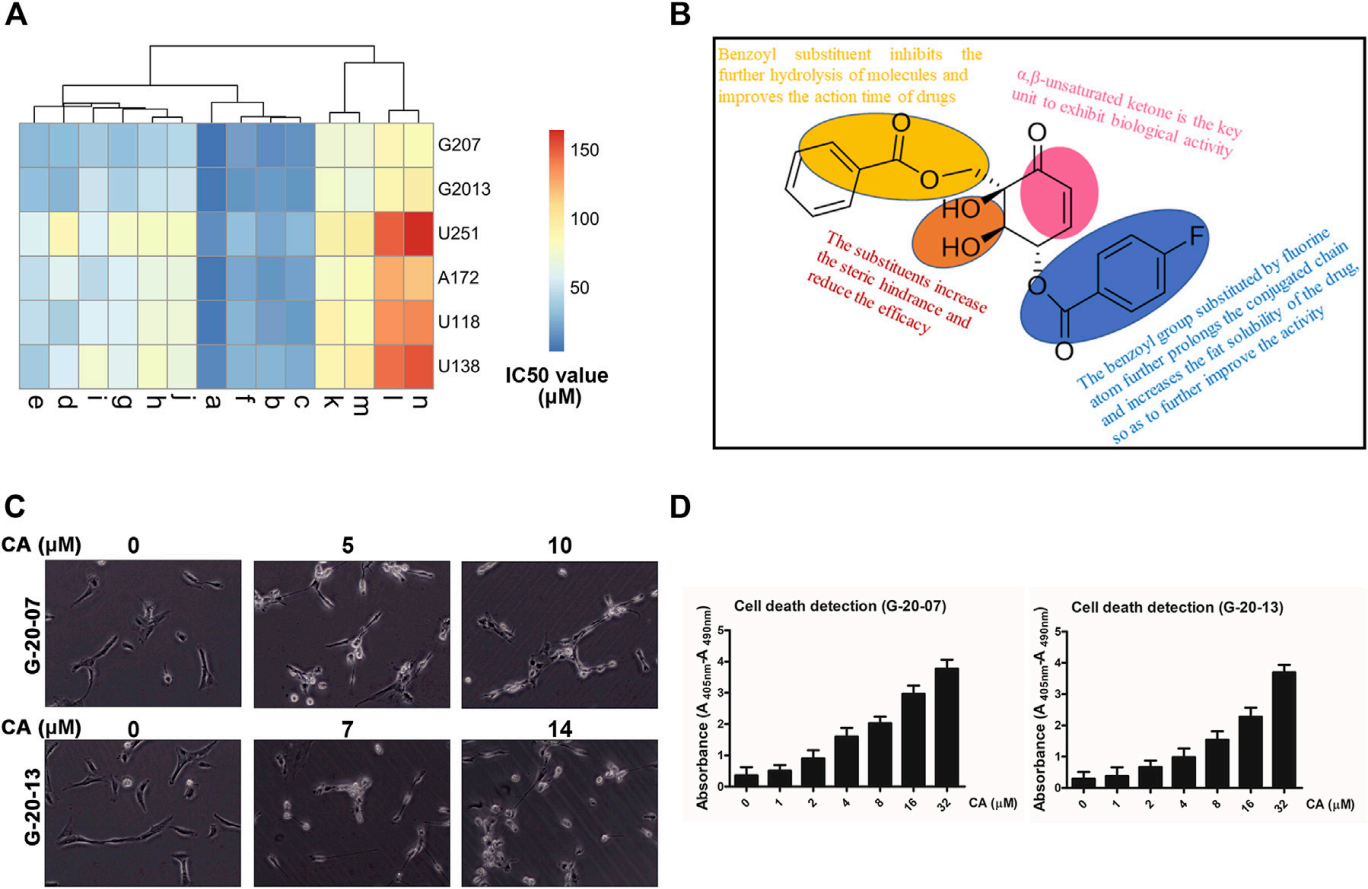
Inhibitory effect of CA on the malignant behaviors of primary GBM cell lines. **(A)** Clustering analysis of the inhibitory effect of a series of (+)-zeylenone analogs on four GBM cell lines and two primary GBM cells. **(B)** Apoptotic change in primary GBM cells after CA treatment that was evaluated using ELISA. **(C)** Morphological change in primary GBM cells after CA treatment. **(D)** Structure–activity relationship analysis of CA ((1R, 2R, 3S)-3-p-fluorobenzoyl-zeylenone).

To investigate the influence of hydroxyls at position C-1/2/3 on the anti-GBM activity, we synthesized a series of (+)-zeylenone analogs **a–n**. The synthesis and structure of these derivatives are outlined in Figure 1. Subsequently, the anti-GBM activities of the derivatives were evaluated using the half inhibitory concentration (IC_50_) value against two primary GBM cells (G-20-07 and G-20-13) and four GBM cells (U251, A172, U118, and U138). As shown in Figure 2A, it was observed that the substituents of hydroxyls at C-1/2 significantly decreased efficacy. Specifically, the IC_50_ values of (+)-zeylenone analogs such as **g, i, k, m** or **h, j, l, n** increased as the molecular weight of substituted groups increased at C-2 or C-1, 2 positions, suggesting that the steric hindrance of substituents has a great impact on the anti-tumor activity. Among these derivatives with substituents at C-3, (+)-zeylenone **d** possessed the weakest activity, implying that the benzoyl unit is indispensable for zeylenone to exert bioactivity.

Moreover, (+)-zeylenone **a** ((1R, 2R, 3S)-3-p-fluorobenzoyl-zeylenone, CA) exhibited the most remarkable inhibitory effect on primary GBM cell viabilities. It can be concluded that the benzoyl group substituted by a fluorine atom can further improve the effect, probably due to prolonging the conjugated chain and increasing the fat solubility of the drug. Thus, these findings indicated that both the ortho-dihydroxy and benzoyl groups are the critical units of zeylenone to exhibit the effect, and a fluorine atom substituent in the benzoyl group could enhance the anti-glioma effect. The relationship between structures and anti-tumor activity of CA is summarized in Figure 2B.

Moreover, G-20-07 and G-20-13 cells showed significant changes in cellular morphologies, such as shrinkage and roundness, after treating with CA (Figure 2C). Similarly, the results of ELISA suggested that CA caused dose-dependent cell death in GBM cells (Figure 2D). The results above suggest that CA exhibits obvious anti-tumor activities in GBM.

### Compound CA treatment suppressed the malignant behaviors of GBM cells

**SCHEME 1:**
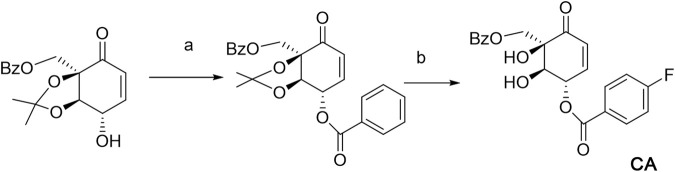
Biosynthesis pathway for CA. **(A)** RCOOH, DMAP, and DCC. **(B)** HCl and MeOH, 90%.

**FIGURE 3:**
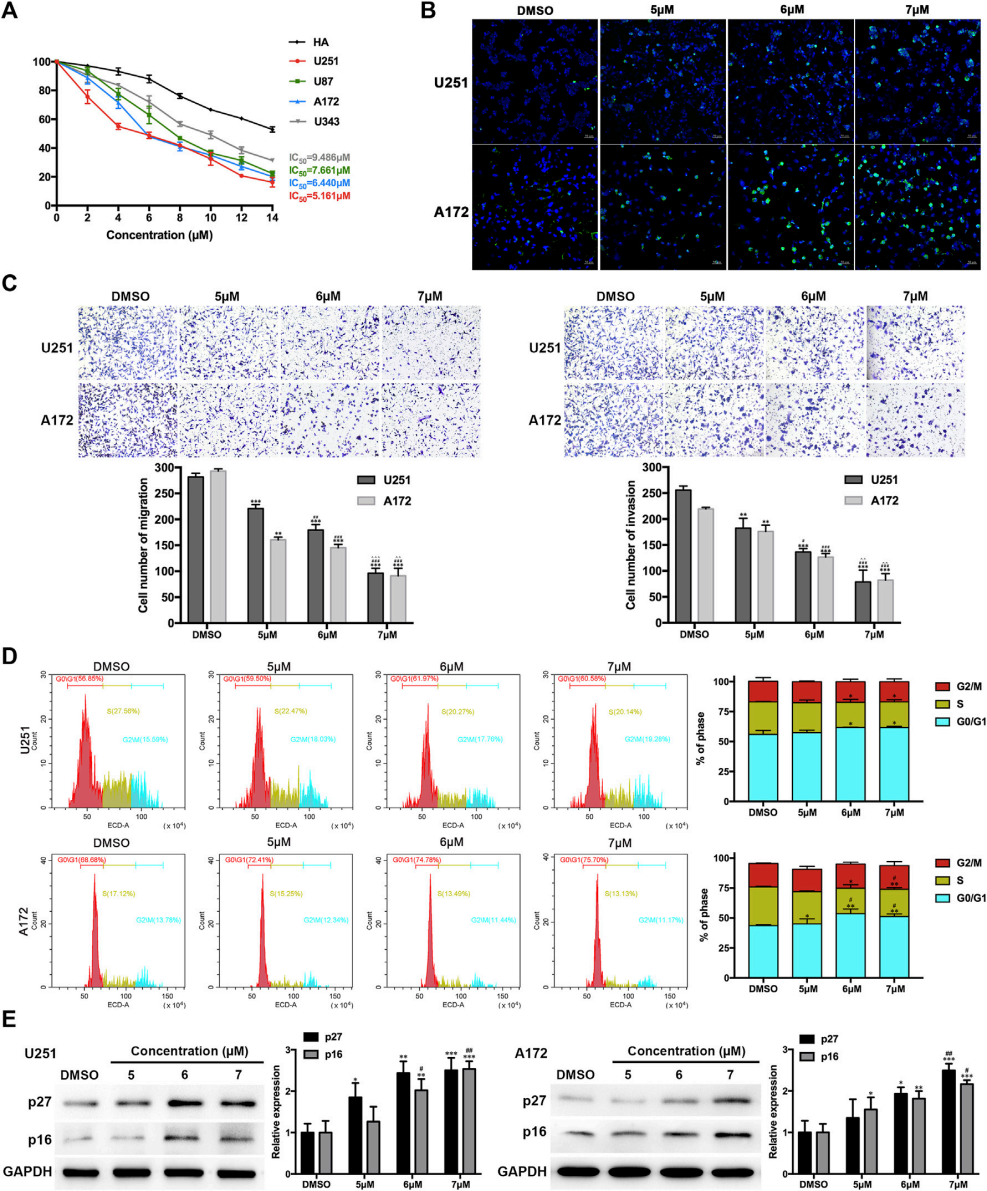
Effects of CA treatment on GBM cells. **(A)** The IC_50_ value of CA in GBM cells and HA was calculated. **(B)** TUNEL staining of GBM cells was carried out. Positive cells are indicated in green, and DAPI counterstain is indicated in blue. **(C)** Migration and invasion of GBM cells were evaluated via transwell assays. **(D)** Cell cycle distribution was analyzed using flow cytometry after CA treatment (0, 5, 6, and 7 µM). **(E)** U251 and A172 cells were treated with different concentrations of CA for 24 h, and the expression levels of p27 and p16 were analyzed. The results are presented as the mean ± SD (n = 3, each group), ^*^ *p* < 0.05 or ^**^ *p* < 0.01 vs. DMSO group. ^#^ *p* < 0.05 or ^##^ *p* < 0.01 vs. 5 µM. ^△△^ *p* < 0.01 vs. 6 µM.

To further elucidate the effects of CA (Scheme 1) on GBM, the half inhibitory concentration (IC_50_) value of CA in GBM cell lines (U251, U87, A172, and U343) and human astrocyte (HA) cells was calculated. As depicted in Figure 3A, the IC_50_ values of CA were notably low in GBM cell lines, particularly in U251 (5.161 µM) and A172 (6.440 µM). Consequently, U251 and A172 were chosen for a more detailed exploration of the effects of CA. TUNEL staining results revealed a gradual increase in the number of DNA-damaged GBM cells with escalating concentrations of CA (Figure 3B). Similarly, CA significantly inhibited the migration and invasion abilities of U251 and A172 at low doses (Figure 3C). To further investigate the anti-tumor effect of CA on GBM cells, we performed cell cycle assays. As shown in Figure 3D, no significant change was observed in the G2/M phase, while low-dose CA treatment distinctly induced G0/G1 arrest of GBM cells, leading to a subsequent decrease in the cell ratio in the S phase. P27^KIP1^ and p16^INK4A^ are recognized for their ability to arrest tumor cells in the G1 phase by interfering with the activities of CDKs, which play key roles in G1/S transitions (Witkiewicz et al., 2022). Consequently, we examined the protein expression of these two CDKIs, revealing that the expression levels of p27 and p16 increased with the escalating concentrations of CA treatment (Figure 3E).

### CA increased the expression of p27 and p16 by reducing the recruitment effect of EZH2 on p27 and p16 through PRC2

**FIGURE 4:**
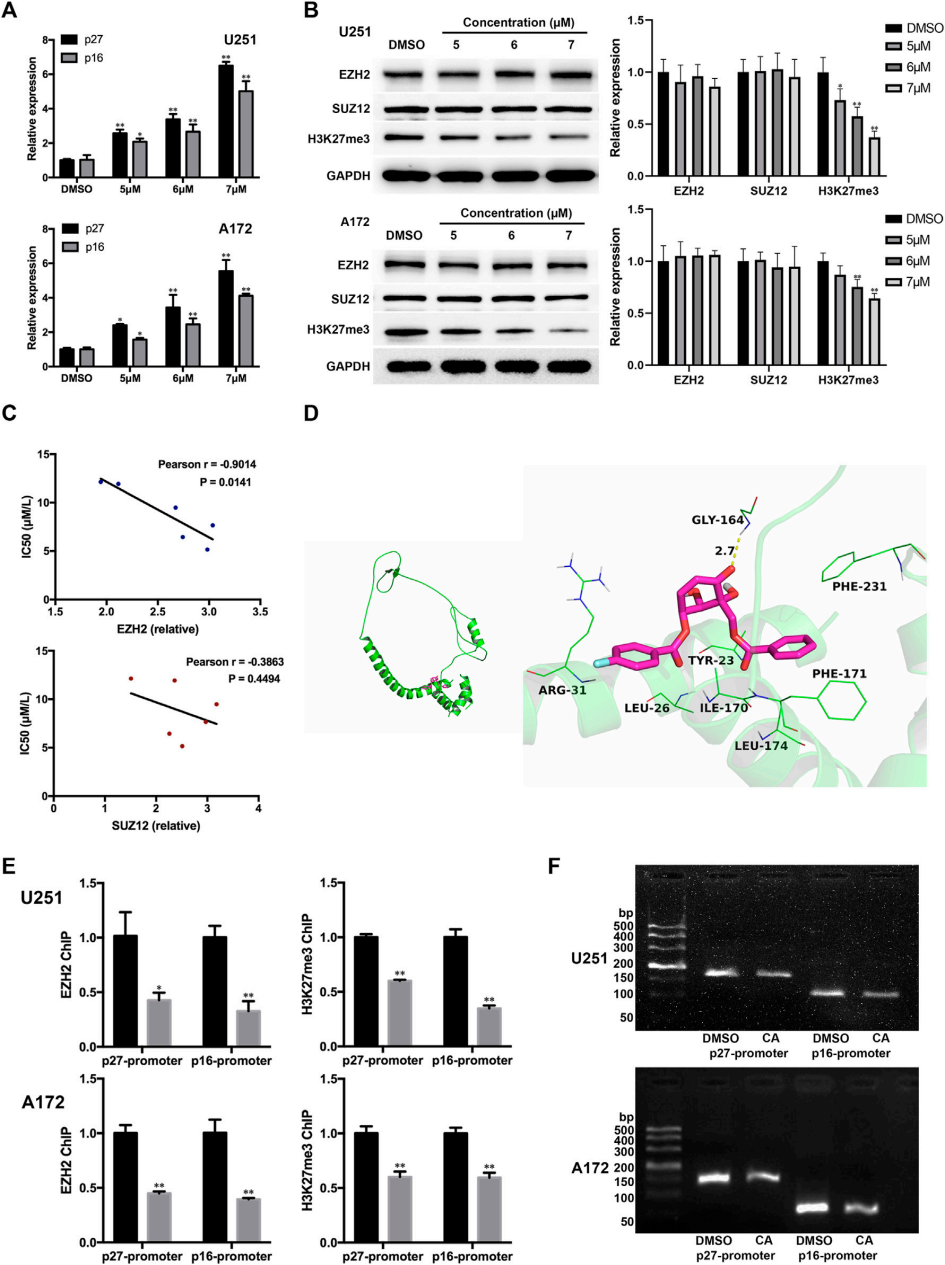
CA treatment affects p27 and p16 expression via interfering with EZH2. **(A)** Effects of CA treatment on the mRNA expression levels of p27 and p16 (n = 3, each group). ^*^ *p* < 0.05 or ^**^ *p* < 0.01 vs. DMSO group. **(B)** Effects of different concentrations of CA treatment on EZH2, SUZ12, and global H3K27me3 expression (n = 3, each group). ^*^ *p* < 0.05 or ^**^ *p* < 0.01 vs. DMSO group. **(C)** Correlation analysis between the IC_50_ value of CA and the expression levels of EZH2 and SUZ12 in GBM cells. **(D)** Molecular docking details of CA and EZH2. **(E)** ChIP-qPCR analyses of p27 and p16 promoter regions with antibodies targeting EZH2 and H3K27me3 in U251 and A172 cells (n = 3, each group). ^*^ *p* < 0.05 or ^**^ *p* < 0.01 vs. anti-IgG group. **(F)** CA treatment reduced the binding between EZH2 and the p27 and p16 promoter regions in U251 and A172 cells.

EZH2 is the key subunit of PRC2, responsible for the methylation of lysine 27 on histone H3 (H3K27), resulting in the repression of gene expression. P27^KIP1^ and p16^INK4A^ are known to be the targets of PRC2 repression. In glioma, some synthetic compounds have been found to induce the G1 phase arrest of tumor cells by inhibiting EZH2 expression and reducing the inhibitory effect of PRC2 on p21, p27, and p16 gene expression (Mohammad et al., 2017; Stazi et al., 2019). In order to further investigate the potential mechanism of CA regulation of GBM cells, we detected mRNAs of p27 and p16. The qPCR results showed that the mRNA expression of both genes increased after CA treatment (Figure 4A). We then surmised whether CA altered p27 and p16 through the same PRC2 mechanism. As shown in Figure 4B, CA treatment had no significant effect on the expression of EZH2 and the core non-catalytic subunit SUZ12. However, the global level of H3K27me3 was obviously decreased with CA treatment. The results of correlation analysis showed that the IC_50_ value of CA in primary, U251, U87, A172, and U343 GBM cells was negatively correlated with the expression level of EZH2 (Figure 4C).

Moreover, CA was docked into the binding site of EZH2, and the results are shown in Figure 4D. The maximum binding affinity between CA and EZH2 was predicted to be −5.8 kcal/mol, and CA adopted a compact conformation to bind to the site of EZH2. CA is located at the hydrophobic pocket, surrounded by the residues Leu-26, Ile-170, Phe-171, Leu-174, and Phe-231, forming a strong hydrophobic binding. Detailed analysis showed that the phenyl group of CA formed the CH–π interactions with the residues Phe-171 and Phe-231. The 4-fluorophenyl group of CA formed the cation–π interaction with the residue Arg-31. Importantly, one key hydrogen bond interaction was observed between CA and the residue Gly-164 (bond length: 2.7 Å), which was the main interaction between CA and EZH2. Combining the above results, we proposed that CA might indirectly interfere in the binding between EZH2 and p27 or p16 genes. To confirm our hypothesis, we carried out the ChIP assays. As shown in Figure 4E, CA treatment significantly reduced the binding of EZH2 or H3K27me3 to p27 and p16 promoter regions in U251 and A172 cells. In addition, the loss of p27 and p16 promoter fragmentations occupied by EZH2 was also observed by agarose gel electrophoresis (Figure 4F). The above results suggest that CA increases the expression of p27 and p16 by reducing the EZH2-mediated inhibitory effect of PRC2 on them.

### CA treatments combined with EZH2 knockdown produced significant tumor inhibition in orthotopic GBM xenograft models

**FIGURE 5:**
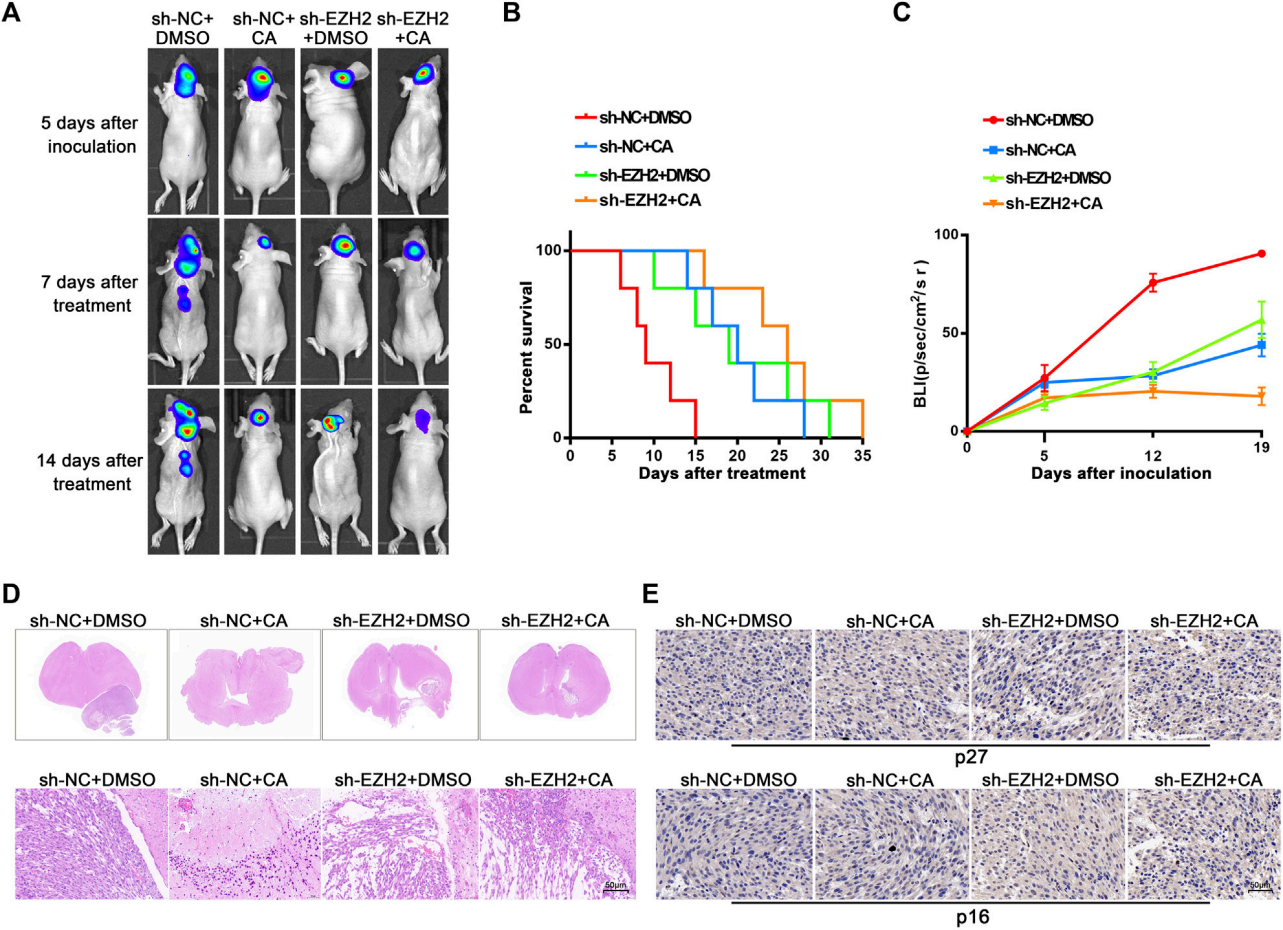
Orthotopic tumor xenograft experiments. **(A)** Bioluminescence images of the nude mice injected with different U251 cells were recorded on day 5 after injection and days 7 and 14 after DMSO (50 μL/2 days) or CA (/10 mg/kg/2 days) treatment, respectively (n = 5, each group). **(B)** Survival curves from the respective mice model. **(C)** The quantitation of bioluminescence imaging was calculated. **(D)** Representative H&E staining scans (upper row) and respective magnified images of tumors that were extracted from different groups (lower row). Arrows indicate the tumor area, and scale bars represent 50 μm. **(E)** The expression of p27 and p16 were detected using immunohistochemistry staining.

EZH2 plays cancer-promoting roles by causing the formation of heterochromatin complexes and initiating gene silencing in cancer cells. As previously mentioned, multiple EZH2 inhibitors have been developed and evaluated in clinical trials (Paskeh et al., 2022). Our above results suggest that CA has a cancer-inhibiting effect by interfering with EZH2. Therefore, we further determined the potential functions of CA *in vivo* combined with EZH2 knockdown. As shown in Figures 5A–C, both CA treatment and EZH2 knockdown showed inhibitory effects on tumorigenesis and prolonged the survival of nude mice models, and knockdown combined with CA treatment led to more remarkable effects. In addition, the H&E staining scan revealed the smallest tumor volume and the least significant tumor tissue heterogeneity in the combination group (Figure 5D). Moreover, immunohistochemistry results demonstrated that the expression levels of p27 and p16 were higher in the CA treatment and the EZH2 knockdown groups, with the highest expression observed in the combination group (Figure 5E). The results above show that CA could enhance the tumor-suppressive effect of EZH2 knockdown in mice models, suggesting that CA could be a potential therapeutic candidate in combination with EZH2 inhibitors.

### CA combined with EZH2 knockdown did not induce obvious toxicity effects in xenograft models

**FIGURE 6:**
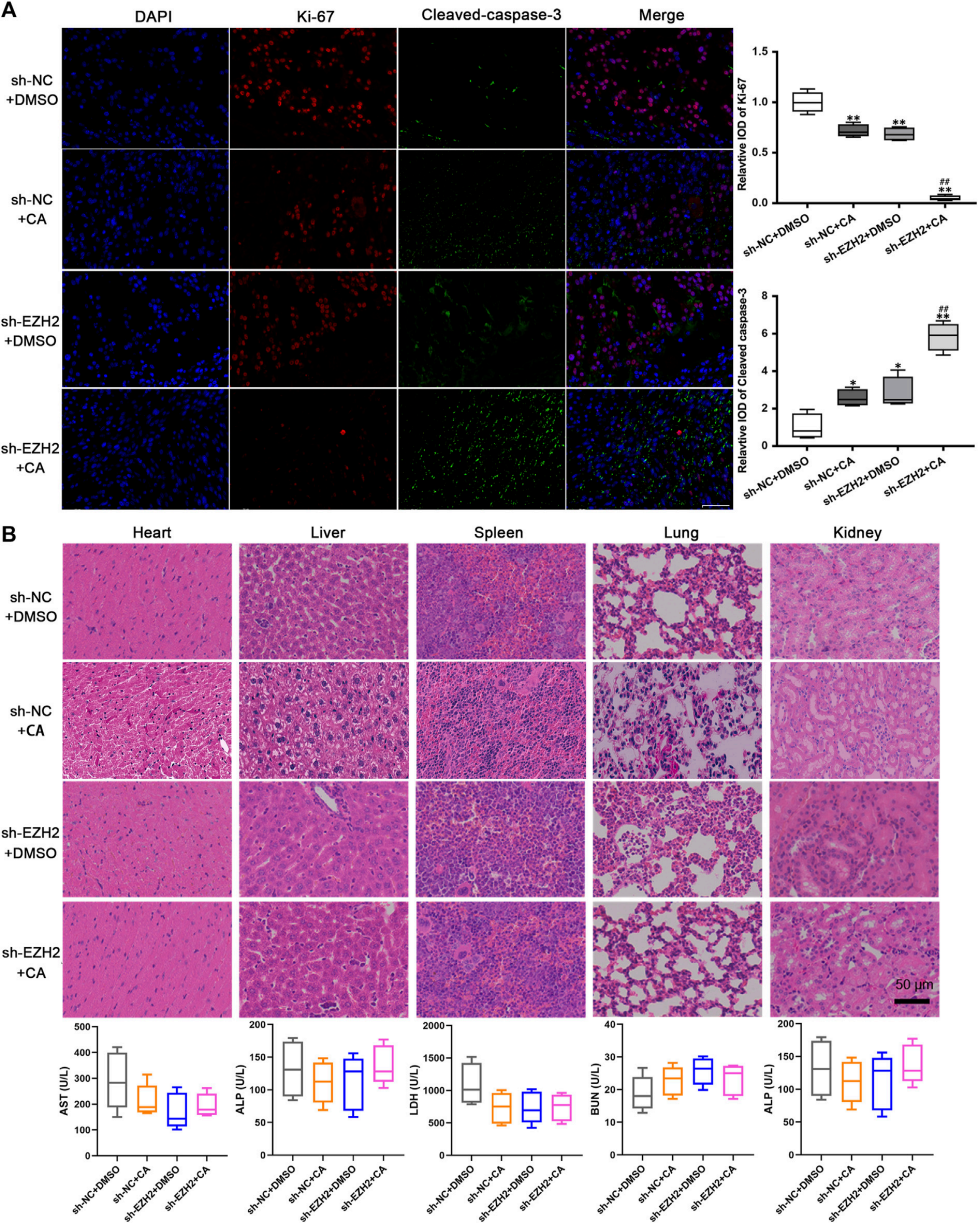
CA exhibited no obvious toxicity effects *in vivo*. **(A)** The expression levels of Ki-67 and cleaved-caspase-3 were observed using immunofluorescence. ^*^ *p* < 0.05 or ^**^ *p* < 0.01 vs. sh-NC + DMSO group. ^##^ *p* < 0.01 vs. sh-EZH2 + DMSO group. **(B)** Toxicity effects of CA on other organs of xenograft models.

The results from immunofluorescence assays revealed that compared with the negative control group, tumors in the CA treatment group, EZH2 knockdown group, and combination group exhibited lower expression of Ki-67 and higher expression of cleaved-caspase-3. Notably, the group with EZH2 knockdown combined with CA treatment demonstrated the lowest Ki-67 expression and the highest cleaved-caspase-3 levels (Figure 6A). These findings indicate that both CA treatment and the combined approach effectively suppressed the proliferation of GBM cells *in vivo* while promoting apoptosis. The toxic effects of CA on the heart, liver, spleen, lungs, and kidneys of mouse models were evaluated using H&E staining and ELISA. As shown in Figure 6B, CA exhibited no remarkable effects on the levels of aspartate aminotransferase (AST), alanine aminotransferase (ALT), lactate dehydrogenase (LDH), blood urea nitrogen (BUN), and alkaline phosphatase (ALP) in these organs, suggesting that CA treatment did not induce notable damage to other organs.

## Discussion

GBM represents the most lethal type of CNS cancer, marked by high mortality rates and limited therapeutic options. In the quest for effective treatments beyond molecular targets, the exploration of potential agents capable of efficiently combating GBM is paramount importance. Increasingly, phytochemicals have been identified for their inhibitory effects on the malignant behaviors of GBM cells by targeting key tumor regulators or selectively interfering with molecular interactions (Santangelo et al., 2021; Wong et al., 2021). In recent years, a number of natural polyoxygenated cyclohexene derivatives, including zeylenone, have been isolated and identified from *U. grandiflora* (He et al., 2021; Cheng et al., 2023).

Since its initial discovery in 1997, zeylenone has consistently demonstrated therapeutic potential against inflammation, diabetes, and various cancers, such as prostate, gastric, and cervical cancers (Quimque et al., 2022). However, due to its multichiral centers and complex structure, zeylenone is somewhat difficult to synthetically obtain and to conformationally study (Vinaykumar and Venkateswara Rao, 2021). A prior study successfully achieved the total syntheses of zeylenone and its derivatives through one-step dihydroxylation from quinic acid, further showing that the hydroxyl group at the C-1/2/3 position of (+)-zeylenone is essential for its anti-tumor activity (Sun et al., 2021). Building upon these findings, our present study involved the synthesis of a series of analogs, providing the first conformational validation of their anti-GBM activities. Notably, (1R, 2R, 3S)-3-p-fluorobenzoyl-zeylenone (C_21_H_17_FNaO_7_ [M + Na]^+^, CA) exhibited the lowest IC_50_ value in GBM cells. This result suggests that substituting the benzoyl group with a fluorine atom significantly enhances the anti-GBM activity of (+)-zeylenone. Consequently, CA was selected and synthesized for further investigation of its potential roles in GBM.

Cancer is well-known for its hallmark feature of continuous and excessive division of cancer cells, a process tightly regulated by various cell cycle mechanisms (Matthews et al., 2022). Treatments that exploit the association between cancer and the cell cycle, widely called “cyclotherapy,” have always attracted the attention of researchers (Johnson et al., 2021). Critical in determining whether cells can enter the cell cycle and subsequently proliferate, the G1 phase precedes two key events: DNA replication and cytokinesis (Zatulovskiy et al., 2020). In alignment with the results obtained from primary GBM cells, our findings demonstrated that CA exhibits a lower IC_50_ in glioma cell lines (U251, U87, A172, and U343) compared to HA, with particularly notable effectiveness in U251 and A172. Moreover, CA treatment inhibited the malignant biological behaviors of GBM cells while obviously eliciting a G0/G1 cell cycle arrest in GBM cells, resulting in a decreased number of cells in the S phase. Additionally, CA treatment led to an increase in the expression levels of p27 and p16. The balance between cyclins or cyclin-dependent kinases (CDKs) and CDKIs regulates the progression and transition of G1 to S phase (Huang et al., 2004). P27^Kip1^ and p16^INK4a^ belong to two major categories of CDKIs: Cip/Kip and INK4, respectively (Sherr and Roberts, 1999). The low levels of p27 and p16, coupled with increased CDK activities, are often associated with the tumorigenesis of various cancers, including GBM (Razavipour et al., 2020; Gkikas et al., 2021; Lu et al., 2022). Our study suggests that CA may induce a G0/G1 cell cycle arrest in GBM cells by modulating the expression levels of p27 and p16.

The mechanism underlying zeylenone-induced tumor progression has been reported in a few instances. For instance, zeylenone inhibited the viability and metastasis of prostate cancer cells via the Wnt/β-catenin signaling pathway (Zeng et al., 2018). Through the mitochondrial apoptosis pathway, zeylenone induced apoptosis in gastric cancer cells and inhibited the tumorigenicity of gastric cancer cells *in vitro* and *in vivo* (Yang et al., 2018). PRC2 is a multi-protein chromatin methyltransferase complex that plays crucial roles in maintaining gene expression and cellular properties. It was found to promote the development of multiple tumors by catalyzing the methylation of lysine 27 on histone H3 (H3K27) (Li et al., 2021). In skeletal muscle cells, PRC2 was found to promote the G1/S transition by directly regulating cyclin D1 and cyclin E1 (Adhikari and Davie, 2020). In the present study, we observed a significant negative correlation between CA IC_50_ and the expression levels of EZH2, the core catalytic component of PRC2 (Eich et al., 2020). EZH2 has been shown to suppress the expression of p27 in hepatocellular carcinoma cells through methylation of the p27 promoter (Hu et al., 2016). Similarly, PRC2 epigenetically suppresses its target gene p16, thereby influencing the cellular lifespan in acute myeloid leukemia cells and mantle cell lymphoma cells (Martin-Perez et al., 2010; Fiskus et al., 2012). Our study revealed that CA treatment affected the total level of H3K27me3, although it did not impact the expression of EZH2 or SUZ12. Through a series of experiments, we further elucidated that CA promotes the expression of p27 and p16 in GBM cells by interfering with EZH2. However, the mechanism through which CA interferes with the function of EZH2 and whether CA selectively affects the regulation of EZH2 on the target still require further investigation.

A study on osteosarcoma demonstrated that zeylenone could synergize with cisplatin, enhancing cisplatin efficacy by inducing DNA damage and cell cycle arrest and activating the AKT/GSK3β signaling pathway (Yang et al., 2021). Meanwhile, considering the crucial role of EZH2 in cancer carcinogenesis and development through PRC2, numerous molecules have been synthesized and developed as potent EZH2 inhibitors, including DZNep, GSK126, tazemetostat, and CPI-169 (Bitler et al., 2015; Jiang et al., 2021). However, their applications are constrained by poor brain penetration owing to the blood–brain barrier (Zhang et al., 2015). Through the establishment of intracranial orthotopic models in nude mice, we observed that CA treatment significantly enhanced the inhibitory effect of EZH2 knockdown on the tumorigenic ability of GBM cells and prolonged the survival of nude mice. Previous findings indicated that zeylenone inhibited tumor cell activity without significant cytotoxicity to normal cells (Yang et al., 2021). Consistently, our study found that CA showed no remarkable toxicity effects on other organs in nude mice. Our *in vivo* results suggest a potential application of CA to synergize the glioma-suppressive effects of EZH2 inhibitors.

**FIGURE 7:**
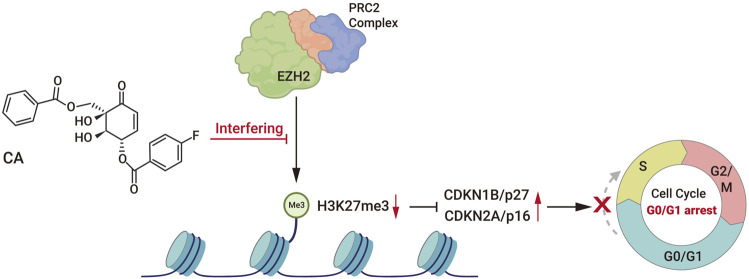
Diagram of the regulatory mechanism of CA.

In summary, we synthesized a series of zeylenone analogs, a naturally occurring polyoxygenated cyclohexenes, and identified that (1R, 2R, 3S)-3-p-fluorobenzoyl-zeylenone (C_21_H_17_FNaO_7_ [M + Na]^+^, CA) exhibits optimal anti-GBM activity among others. This study is the first to elucidate the molecular mechanism through which CA induces the G0/G1 phase arrest in GBM cells by interfering with EZH2, thereby alleviating the inhibitory effect of PRC2 on p27 and p16 (Figure 7). Furthermore, we substantiated the role of CA in inhibiting the tumorigenicity of GBM cells *in vivo* and ensured its safety through *in vivo* experiments. Our findings reveal the anti-GBM activity of CA, a derivative of (+)-zeylenone, indicating its potential clinical application value and offering novel therapeutic insights into GBM.

## Data Availability

The raw data supporting the conclusion of this article will be made available by the authors without undue reservation.
