# Supplementary material for: Cyclohexene oxide CA, a derivative of zeylenone, exhibits anti-cancer activity in glioblastoma by inducing G0/G1 phase arrest through interference with EZH2
Source: Front Pharmacol. 2024 Jan 9;14:1326245. doi: 10.3389/fphar.2023.1326245 (PMC10803536; doi:10.3389/fphar.2023.1326245)
Supplement: Supplementary file 1 [file Presentation1.ZIP › Additional File 1.docx]

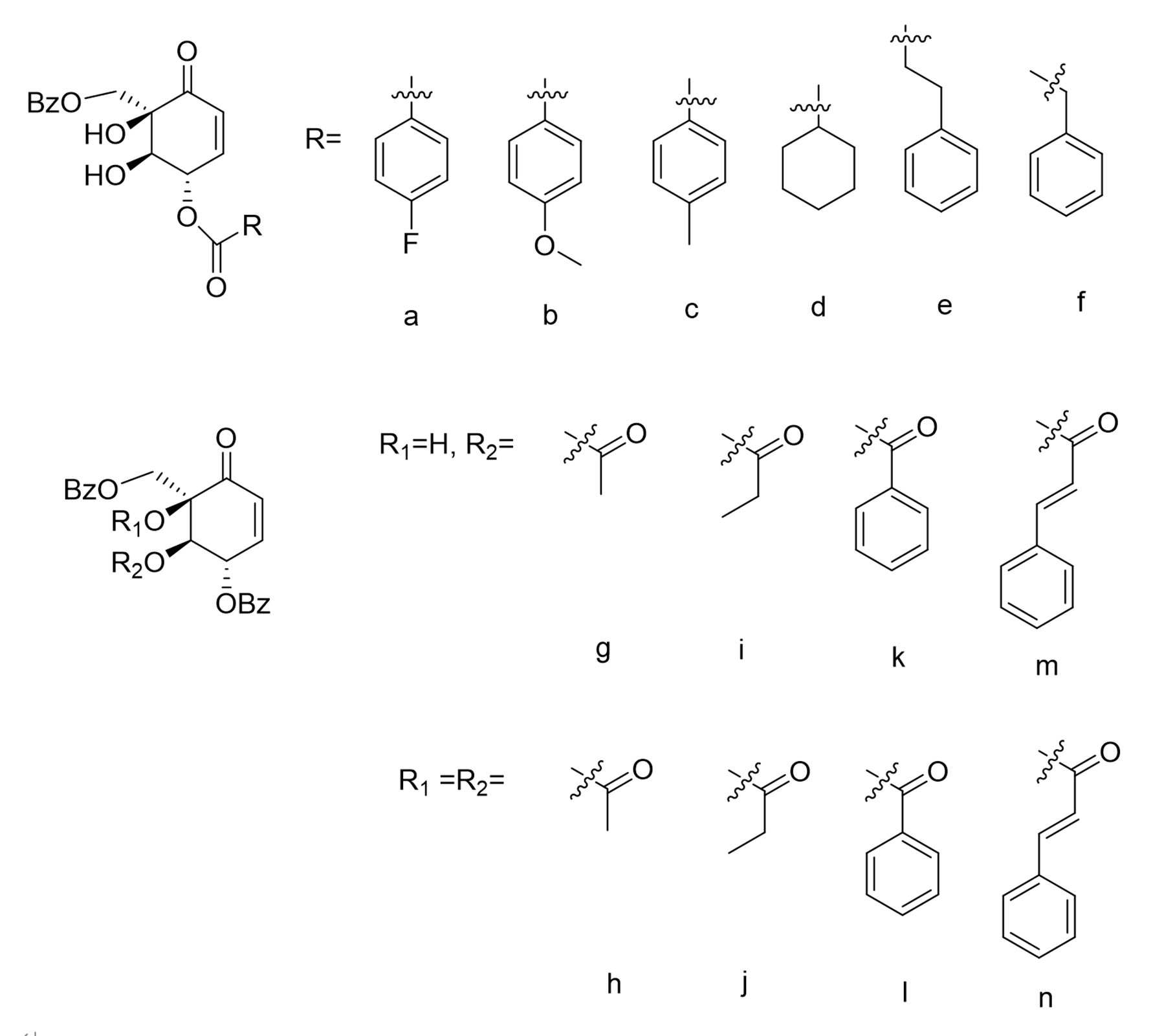


Synthesis of **a-f**

Zeylenone was first protected by 2,2-dimethoxypropane, then dissolved in 5 ml DCM, triethylamine (0.6 ml, 4 mmol), DMAP (25 mg, 0.2 mmol), and benzoyl chloride (360 μ L, 3mmol)) were added successively. After stirring for 2h at RT, the reaction was quenched with saturated sodium bicarbonate solution, extracted with DCM, washed with water, dried (Na_2_SO_4_), and concentrated under reduced pressure. Crude product was purified by silica gel column chromatography to give **a-f.**

**(a)** (*1R, 2R, 3S*)-3-*p*-fluorobenzoyl-zeylenone: HPLC Rt=17.795 min; White solid; Mp 152-154 °C; [α]^20^ D =+18.0 (c = 0.3, CHCl_3_); ^1^HNMR (600 MHz, CDCl_3_): δ (ppm)= 8.03-8.01 (m, 2H, Ar-H), 7.93-7.92 (m, 2H, Ar-H), 7.56 (t, *J* = 7.4 Hz, 1H, Ar-H), 7.41 (t, *J* = 7.8 Hz, 2H, Ar-H), 7.08 (t, *J* = 8.6 Hz, 2H, Ar-H), 6.95 (ddd, *J* = 10.2, 4.1, 1.2 Hz, 1H, H-4), 6.34 (d, *J* = 10.2 Hz, 1H, H-5), 5.94 (m, 1H, H-3), 4.80 (d, *J* = 11.5 Hz, 1H, H-7a), 4.59 (d, *J* = 11.5 Hz, 1H, H-7b), 4.37 (s, 1H, H-2), 4.10 (s, OH), 3.24 (s, OH); ^13^CNMR (151 MHz, CDCl_3_): δ (ppm)= 196.3 (C-1), 166.8 (d, *J_C-F_*= 253.5.Hz, C-F), 166.4(C=O), 164.6 (C=O), 142.8 (C-4), 133.7 (Ar-C), 132.6 (Ar-C), 132.6 (Ar-C), 129.9 (2🞨Ar-C), 129.2 (Ar-C), 128.8 (C-5), 128.6 (2 🞨 Ar-C), 125.1 (Ar-C), 116.1 (Ar-C), 116.0 (Ar-C), 77.4 (C-2), 71.7 (C-1), 69.5 (C-3), 65.5 (C-7); HRMS (ESI)m/z calcd. C_21_H_17_FNaO_7_ [M + Na]^+^: 423.0857, found 423.0856.

**(b)** (*1R, 2R, 3S*)-3-*p*-methoxybenzoyl-zeylenone: HPLC Rt=18.012 min White solid;Mp 186-187°C; [α]^20^ D =-22.9 (c = 0.5, CHCl_3_); ^1^HNMR (600 MHz, CDCl_3_): δ (ppm)= 7.96 (d, *J* = 9.0 Hz, 2H, Ar-H), 7.94 (dd, *J* = 8.3, 1.2 Hz, 2H, Ar-H), 7.55 (t, *J* = 7.4 Hz, 1H, Ar-H), 7.41 (dd, *J* = 8.1, 7.6 Hz, 2H, Ar-H), 6.96 (ddd, *J* = 10.2, 4.2, 1.5 Hz, 1H, H-4), 6.89 (d, *J* = 9.0 Hz, 2H, Ar-H), 6.33 (dd, *J* = 10.2, 0.9 Hz, 1H, H-5), 5.95-5.90 (m, 1H, H-3), 4.84 (d, *J* = 11.5 Hz, 1H, H-7a), 4.59 (d, *J* = 11.5 Hz, 1H, H-7b), 4.36 (dd, *J* = 3.3, 1.5 Hz, 1H, H-2), 4.11 (s, OH), 3.85 (s, 3H, OMe), 3.18 (s, OH); ^13^CNMR (151 MHz, CDCl_3_): δ (ppm)= 196.6 (C-6), 166.3 (C=O), 165.2 (C=O), 164.1 (C=C(OMe)), 143.1 (C-4), 139.9 (Ar-C), 133.6 (Ar-C), 132.1 (2🞨Ar-C), 129.9(2🞨Ar-C), 129.3 (Ar-C), 128.6 (2🞨Ar-C), 128.6 (C-5), 121.1 (Ar-C), 114.1 (2🞨Ar-C), 77.4 (C-2), 71.8 (C-1), 69.0 (C-3), 65.7 (C-7), 55.7 (OMe); HRMS (ESI): m/z calcd. C_22_H_20_NaO_8_ [M + Na]^+^: 435.1056, found 435.1080.

**(c)** (*1R, 2R, 3S*)-3-*p*-methylbenzoyl-zeylenone: HPLCRt=17.916 min;White solid; Mp 148-150 °C; [α]^20^ D =-14.9 (c = 0.2, CHCl_3_); ^1^HNMR (600 MHz, CDCl_3_): δ (ppm)= 7.94 (d, *J* = 7.5 Hz, 2H, Ar-H), 7.90 (d, *J* = 8.1 Hz, 2H, Ar-H), 7.55 (t, *J* = 7.4 Hz, 1H, Ar-H), 7.41 (t, *J* = 7.7 Hz, 2H, Ar-H), 7.21 (d, *J* = 8.0 Hz, 2H, Ar-H), 6.96 (dd, *J* = 10.1, 3.9 Hz, 1H, H-4), 6.33 (d, *J* = 10.2 Hz, 1H, H-5), 5.94 (t, *J* = 3.5 Hz, 1H, H-3), 4.84 (d, *J* = 11.5 Hz, 1H, H-7a), 4.59 (d, *J* = 11.5 Hz, 1H, H-7b), 4.37 (d, *J* = 2.2 Hz, 1H, H-2), 2.39 (s, 3H, Me-Ph); ^13^CNMR (151 MHz, CDCl_3_): δ (ppm)= 196.5 (C-6), 166.3 (C=O), 165.6 (C=O), 144.9 (C-4), 143.0 (C=CMe), 133.6 (Ar-C), 130.0 (2🞨Ar-C), 129.9(2🞨Ar-C), 129.5 (2🞨Ar-C), 129.3 (Ar-C), 128.7 (C-5), 128.6 (2🞨Ar-C), 126.1 (Ar-C), 77.4 (C-2), 71.8 (C-1), 69.2 (C-3), 65.6 (C-7), 21.9 (Ar-Me); HRMS (ESI): m/z calcd. C_22_H_20_NaO_7_ [M + Na]^+^: 419.1107, found 419.113.

**(d)** (*1R, 2R, 3S*)-3-(cyclohexanecarbonyl)-zeylenone: HPLC Rt =22.584 min; White solid; Mp 141-142°C; [α]^20^ D =+33.8 (c = 0.2, CHCl_3_); ^1^H-NMR (600 MHz, CDCl_3_): δ (ppm)= 7.99 (dd, *J* = 8.3, 1.2 Hz, 2H, Ar-H), 7.58 (t, *J* = 7.4 Hz, 1H, Ar-H), 7.44 (t, *J* = 7.8 Hz, 2H, Ar-H), 6.82 (ddd, *J* = 10.2, 4.2, 1.4 Hz, 1H, H-4), 6.28 (dd, *J* = 10.2, 0.8 Hz, 1H, H-5), 5.69 (dd, *J* = 5.3, 2.0 Hz, 1H, H-3), 4.71 (d, *J* = 11.6 Hz, 1H, H-7a), 4.52 (d, *J* = 11.6 Hz, 1H, H-7b), 4.18 (dd, *J* = 3.2, 1.4 Hz, 1H, H-2), 4.05 (br s, OH), 3.07 (br s, OH), 2.39-2.30 (m, 1H, CHCO(CH_2_)_2_), 1.98-1.84 (m, 2H, H of cyclohexyl), 1.77-1.63 (m, 3H, H of cyclohexyl), 1.44 (qd, *J* = 13.0, 3.5 Hz, 2H, H of cyclohexyl), 1.24 (ddd, *J* = 24.6, 12.2, 5.8 Hz, 3H, H of cyclohexyl); ^13^CNMR (151 MHz, CDCl_3_): δ (ppm)= 196.5 (C-1), 174.9 (C=O), 166.3 (C=O), 143.0 (C-4), 133.7 (Ar-C), 129.9 (C-5), 129.3 (2🞨Ar-C)), 128.7 (Ar-C), 128.4 (2🞨Ar-C), 77.3 (C-2), 71.8 (C-1), 68.4 (C-3), 65.7 (C-7), 42.1 (C of cyclohexyl), 29.1 (C of cyclohexyl), 29.1 (C of cyclohexyl), 25.7 (C of cyclohexyl), 25.4 (2🞨C of cyclohexyl); HRMS (ESI):m/z calcd. C_21_H_24_NaO_7_ [M + Na]^+^: 411.1420, found 411.1441.

**(e)** (*1R, 2R, 3S*)-3-(phenylpropanecarbonyl)-zeylenone: HPLC Rt =18.105 min; White solid; Mp 171-172°C; [α]^20^ D =+20.6 (c = 0.3, CHCl_3_); ^1^HNMR (600 MHz, CDCl_3_): δ (ppm)=8.03-7.95 (m, 2H, Ar-H), 7.58 (t, *J* = 7.4 Hz, 1H, Ar-H), 7.44 (t, *J* = 7.7 Hz, 2H, Ar-H), 7.28 (t, *J* = 7.5 Hz, 2H, Ar-H), 7.20 (t, *J* = 7.4 Hz, 1H, Ar-H), 7.16 (d, *J* = 7.2 Hz, 2H, Ar-H), 6.71 (dd, *J* = 10.2, 3.9 Hz, 1H, H-4), 6.23 (d, *J* = 10.2 Hz, 1H, H-5), 5.68 (dd, *J* = 5.2, 2.4 Hz, 1H, H-3), 4.61 (d, *J* = 11.5 Hz, 1H, H-7a), 4.45 (d, *J* = 11.5 Hz, 1H, H-7b), 4.08 (d, *J* = 3.6 Hz, 1H, H-2), 4.08 (s, OH), 3.18 (s, OH), 2.95 (dd, *J* = 11.7, 4.5 Hz, 2H, CH_2_CH_2_-Ph), 2.69 (td, *J* = 7.7, 3.6 Hz, 2H, CH_2_CH_2_-Ph); ^13^CNMR (151 MHz, CDCl_3_): δ (ppm)= 196.2 (C-1), 171.9 (C=O), 166.3 (C=O), 143.0 (C-4), 139.9 (Ar-C), 133.7 (Ar-C), 129.9(2🞨Ar-C), 129.3 (Ar-C), 128.7 (2🞨Ar-C)), 128.7 (2🞨Ar-C), 128.4 (2🞨Ar-C), 128.4 (C-5), 126.7 (Ar-C), 77.2 (C-2), 71.7 (C-1), 69.0 (C-3), 65.2 (C-7), 35.8 (CH_2_), 31.0(CH_2_); HRMS (ESI): m/z calcd. C_23_H_22_NaO_7_ [M + Na]^+^: 433.1263, found 433.1275.

**(f)** (*1R, 2R, 3S*)-3-(phenethylcarbonyl)-zeylenone: HPLC Rt =19.215 min; White solid; Mp 148-150°C; [α]^20^ D =+15.0 (c = 0.5, CHCl_3_); ^1^HNMR (600 MHz, CDCl_3_): δ (ppm)= 7.98 (d, J = 7.1 Hz, 1H, Ar-H), 7.59 (t, J = 7.5 Hz, 1H, Ar-H), 7.45 (dd, J = 8.1, 7.6 Hz, 1H, Ar-H), 7.35-7.28 (m, 1H, Ar-H), 7.28-7.22 (m, 3H, Ar-H), 6.79 (ddd, J = 10.2, 4.1, 1.4 Hz, 1H, H-4), 6.26 (dd, J = 10.2, 0.9 Hz, 1H, H-5), 5.72-5.67 (m, 1H, H-3), 4.61 (d, J = 11.6 Hz, 1H, H-7a), 4.35 (d, J = 11.6 Hz, 1H, H-7b), 4.14-4.09 (m, 1H, H-2), 4.03 (s, 1H, OH), 3.73-3.63 (m, 2H, CH_2_-Ph), 3.10 (s, 1H, OH).^13^CNMR (151 MHz, CDCl_3_) δ 196.4(C-6), 170.5 (C=O), 166.3 (C=O), 142.6 (C-4), 133.8 (Ar-C), 133.2 (Ar-C),130.0 (2🞨Ar-C), 129.5(2🞨Ar-C), 129.5 (Ar-C), 129.1 (2🞨Ar-C), 128.8 (2🞨Ar-C), 128.7 (C-5), 127.8 (Ar-C), 77.4 (C-2), 71.8 (C-1), 69.2 (C-3), 65.5 (C-7), 41.4 (CH_2_(CO)Ar).

Synthesis of **g-n**

**g** and **h**: (+)-Zeylenone (40 mg, 0.11 mmol) was dissolved in 2 ml DCM, then triethylamine (15 μl, 0.1 mmol), DMAP (1.5 mg, 0.1 mmol), and acetyl chloride (15.5 μL, 0.22 mmol) were added successively. After stirring for 2 h at RT, the reaction was quenched with saturated sodium bicarbonate solution, extracted with DCM, washed with saturated sodium chloride, dried (Na_2_SO_4_), and concentrated under reduced pressure. Crude product was purified by silica gel column chromatography (petroleum ether: ethyl acetate, 6:1) to give **g** (11 mg, 25%), and (petroleum ether: ethyl acetate, 4:1) to give **h** (27 mg, 71%).

**(g)** (*1R, 2R, 3S*)-2-acetyl-zeylenone: HPLC Rt =22.618 min; White solid; Mp 132-134°C; [α]^20^ D =+33.5 (c = 0.3, CHCl_3_); ^1^HNMR (500 MHz, CDCl_3_) δ 8.01 (dd, *J*= 8.2, 1.1 Hz, 2H), 7.99 (d, *J*=8.0, 1.1 Hz, 2H), 7.59-7.55 (m, 2H), 7.44 (t, *J* = 7.6 Hz, 4H), 7.02 (ddd, *J* = 10.3, 3.6, 0.9 Hz, 1H), 6.38 (dd, *J* = 10.3, 1.1 Hz, 1H), 5.95-5.93 (m, 1H), 5.82 (dd, *J* = 4.4, 0.8 Hz, 1H), 4.80 (d, *J* = 11.4 Hz, 1H), 4.71 (d, *J* = 11.4 Hz, 1H), 3.76 (s, 1H, OH), 2.09 (s, 3H); ^13^CNMR (125 MHz, CDCl_3_) δ 195.00, 169.52, 166.19, 165.39, 143.27, 134.09, 133.62, 130.05, 129.95, 129.33, 129.08, 128.91, 128.69, 76.27, 71.71, 68.77, 65.20, 20.80;HRMS (ESI): m/z calcd. C_243_H_20_NaO_8_ [M + Na]^+^: 447.1056, found 447.1056

**(h)** (*1R, 2R, 3S*)-1,2-diacetyl-zeylenone: HPLC Rt =26.596 min; White solid; Mp 129-130°C; [α]^20^ D =+12.05 (c = 0.1, CHCl_3_); ^1^HNMR (500 MHz, CDCl_3_) δ 8.00 (dd, *J* = 8.3, 1.1 Hz, 2H), 7.98(m, 2H), 7.57 (dd, *J* = 6.8, 2.4 Hz, 2H), 7.45 (dd, *J* = 7.7, 2.3 Hz, 2H), 6.87 (dd, *J* = 10.5, 2.1 Hz, 1H), 6.32 (dd, *J* = 10.5, 2.3 Hz, 1H), 6.18 (d, *J* = 8.3 Hz, 1H), 5.92 (d, *J* = 8.3 Hz, 1H), 4.89 (d, *J* = 10.9 Hz, 1H), 4.64 (d, *J* = 11.0 Hz, 1H), 2.18 (s, 3H), 2.01 (s, 3H); ^13^CNMR (125 MHz, CDCl_3_) δ 190.19 (s), 169.61 (s), 166.19 (s), 165.99 (s), 165.65, 143.87, 133.98, 133.60, 130.09, 129.97, 129.41, 129.35, 128.99, 128.88, 128.69, 80.82, 71.39, 70.82, 61.73, 20.81, 20.72;HRMS (ESI): m/z calcd. C_25_H_22_NaO_9_ [M + Na]^+^: 489.1162, found 489.1158.

**i** and **j** were synthesized by the same method as **g** and **h**, except acetyl chloride changed to propionyl chloride.

**(i)** (*1R, 2R, 3S*)-2-propionyl-zeylenone (10 mg, 24%): HPLC Rt =23.008 min; White solid; Mp 155-157°C; [α]^20^ D =+16.3 (c = 0.1, CHCl_3_); ^1^HNMR (600 MHz, CDCl_3_) δ 8.00 (d, *J* = 7.3 Hz, 2H), 7.96 (d, *J* = 7.3 Hz, 2H), 7.56 (d, *J* = 7.5 Hz, 2H), 7.41 (td, *J* = 7.8, 3.7 Hz, 4H), 7.00 (dd, *J* = 10.2, 3.7 Hz, 1H), 6.37 (d, *J* = 10.2 Hz, 1H), 5.90 (t, *J* = 3.9 Hz, 1H), 5.81 (d, *J* = 4.2 Hz, 1H), 4.78 (d, *J* = 11.4 Hz, 1H), 4.67 (d, *J* = 11.4 Hz, 1H), 3.59 (s, 1H, OH), 2.34 (dd, *J* = 7.4, 6.4 Hz, 2H), 1.08 (t, *J* = 7.5 Hz, 3H); ^13^CNMR (151 MHz, CDCl_3_) δ 195.04 , 169.44 , 166.15, 165.30 , 143.06 , 134.05 , 133.59 , 129.28 , 129.06 , 128.87 , 128.65 , 20.85.

**(j)** (*1R, 2R, 3S*)-1,2-dipropionyl-zeylenone (30 mg, 71%): HPLC Rt =25.772 min; White solid; Mp 155-156°C; [α]^20^ D =-18.8 (c = 0.1, CHCl_3_); ^1^HNMR (600 MHz, CDCl_3_) δ 7.99 (dd, *J* = 7.8, 1.2 Hz, 2H), 7.97 (dd, *J* = 7.8, 1.2 Hz, 2H),7.58-7.56 (m, 2H), 7.44 (m, 4H), 6.88 (dd, *J* = 10.5, 2.0 Hz, 1H), 6.33 (dd, *J* = 10.5, 2.3 Hz, 1H), 6.19 (dd, *J* = 8.3, 2.1 Hz, 1H), 5.95 (d, *J* = 8.4 Hz, 1H), 4.88 (d, *J* = 10.8 Hz, 1H), 4.60 (d, *J* = 10.9 Hz, 1H), 2.46 (q, *J* = 7.5 Hz, 2H), 2.30-2.25 (m, 2H), 1.17 (t, *J* = 7.5 Hz, 3H), 0.96 (t, *J* = 7.5 Hz, 3H); ^13^CNMR (151 MHz, CDCl_3_) δ 190.31, 173.10, 173.06, 165.94, 165.58, 143.97, 133.97, 133.53, 130.04, 129.91, 129.38, 129.06, 128.95, 128.63, 80.47, 71.12, 70.83, 61.96, 27.43, 27.32, 9.12, 9.07;HRMS (ESI): m/z calcd. C_27_H_26_NaO_9_ [M + Na]^+^: 517.1475, found 517.1472.

**k** and **l** were synthesized by the same method as **g** and **h**, except acetylchloride changed to benzoyl chloride.

**(k)** (*1R, 2R, 3S*)-2-benzoyl-zeylenone (12 mg, 20%): HPLC Rt =21.883 min; White solid; Mp 176-177°C; [α]^20^ D =-24.5 (c = 0.3, CHCl_3_); ^1^HNMR (600 MHz, CDCl_3_) δ 8.03 (d, *J* = 7.3 Hz, 2H), 7.98 (d, *J* = 7.5 Hz, 4H), 7.60-7.56 (m, 3H), 7.45-7.40 (m, 6H), 7.09 (ddd, *J* = 10.3, 3.4, 1.8 Hz, 1H), 6.49 (d, *J* = 10.3 Hz, 1H), 6.05 (d, *J* = 3.7 Hz, 2H), 4.93 (d, *J* = 11.5 Hz, 1H), 4.76 (d, *J* = 11.5 Hz, 1H), 3.79 (s, 1H, OH); ^13^CNMR (125 MHz, CDCl_3_) δ 195.65, 166.26, 165.22, 165.06,142.77, 134.03, 133.83, 133.54, 130.10, 130.02, 129.90, 129.24, 129.17, 128.97, 128.84, 128.70, 128.61, 76.54, 72.36, 68.41, 66.13;HRMS (ESI): m/z calcd. C_28_H_22_NaO_8_ [M + Na]^+^: 509.1212, found 509.1208.

**(l)** (*1R, 2R, 3S*)-1,2-dibenzoyl-zeylenone (33 mg,64%): HPLC Rt =26.257 min; White solid; Mp 165-167°C; [α]^20^ D =+10.9 (c = 0.1, CHCl_3_); ^1^HNMR (600 MHz, CDCl_3_) δ 8.09 (dd, *J* = 8.1, 1.2 Hz, 2H), 7.97 (dd, *J* = 8.1, 1.2 Hz, 2H), 7.89 (dd, *J* = 8.3, 1.2 Hz, 2H), 7.87 (dd, *J* = 8.1, 1.2 Hz, 2H),7.64 (m, 1H), 7.53-7.52 (m, 5H), 7.37 (d, 7.9 Hz, 2H), 7.33 (d, 7.8 Hz, 2H),7.29 (d, *J* = 8.1 Hz, 2H), 6.99 (dd, *J* = 10.4, 2.3 Hz, 1H), 6.46 (dd, *J* = 10.2, 7.9 Hz, 2H), 6.32 (d, *J* = 8.1 Hz, 1H), 5.05 (d, *J* = 11.0 Hz, 1H), 4.83 (d, *J* = 11.0 Hz, 1H); ^13^CNMR (125 MHz, CDCl_3_) δ 190.26, 165.97, 165.56, 165.20, 165.12,144.27, 134.27, 133.80, 133.39, 130.34, 130.3,129.91, 129.55, 128.96, 128.92, 128.71, 128.65, 128.62, 128.52, 128.44, 80.62, 72.10, 70.94, 62.98;HRMS (ESI): m/z calcd. C_35_H_26_NaO_9_ [M + Na]^+^: 613.1475, found 613.1470.

**m** and **n** were synthesized synthesized by the same method as **g** and**h**, except changed acetyl chloride to cinnamoyl chloride.

**(m)** (*1R, 2R, 3S*)-2-cinnamoyl-zeylenone (15 mg, 18%): HPLC Rt =20.863 min; White solid; Mp 161-163°C; [α]^20^ D =-25.8 (c = 0.1, CHCl_3_); ^1^HNMR (600 MHz, CDCl_3_) δ 8.03 (dd, *J* =7.3 Hz, 2H), 8.01 (dd, *J* =7.3 Hz, 2H),7.71 (d, *J* = 16.0 Hz, 1H), 7.60-7.54 (m, 2H), 7.50 – 7.49 (m, 2H), 7.42 (m, 8H), 7.07 (dd, *J* = 10.3, 3.7 Hz, 1H), 6.45-6.42 (d, *J* = 10.2 Hz, 1H), 6.42 (d, *J* = 16.0 Hz, 1H), 6.04 (dd, *J* = 5.7, 2.2 Hz, 1H), 5.96 (d, *J* = 4.4 Hz, 1H), 4.87 (d, *J* = 11.4 Hz, 1H), 4.76 (d, *J* = 11.4 Hz, 1H), 3.78 (s, 1H, OH); ^13^CNMR (151 MHz, CDCl_3_) δ 195.07, 166.11, 165.28, 165.20, 146.88, 143.08, 133.93, 133.90,133.40, 130.77, 129.89, 129.80, 128.94, 128.93, 128.70, 128.70, 128.48, 128.34, 128.14, 116.43, 76.24, 71.80, 68.58, 65.50;HRMS (ESI): m/z calcd. C_30_H_42_NaO_8_ [M + Na]^+^: 535.1369, found 535.1367.

**(n)** (*1R, 2R, 3S*)-1,2-dicinnamoyl-zeylenone (37 mg, 68%): HPLC Rt =25.274 min; White solid; Mp 188-190°C; [α]^20^ D =-37.9 (c = 0.3, CHCl_3_); ^1^HNMR (600 MHz, CDCl_3_) δ 8.01 (m, 4H), 7.80 (d, *J* = 16.0 Hz, 1H), 7.62 (d, *J* = 16.0 Hz, 1H), 7.59 – 7.55 (m, 4H), 7.55 – 7.50 (m, 9H), 7.34 (d, *J* = 7.2 Hz, 1H), 7.30 (t, *J* = 7.3 Hz, 2H), 6.94 (dd, *J* = 10.5, 2.0 Hz, 1H), 6.55 (d, *J* = 16.0 Hz, 1H), 6.39 – 6.36 (m, 3H), 6.15 (d, *J* = 8.3 Hz, 1H), 4.99 (d, *J* = 11.0 Hz, 1H), 4.76 (d, *J* = 11.0 Hz, 1H); ^13^CNMR (151 MHz, CDCl_3_) δ 190.31, 165.90, 165.45, 165.40, 165.35, 147.89, 146.93, 143.91, 133.82, 133.81, 133.68, 133.27, 131.07, 130.76, 129.94, 129.86, 129.35, 129.05, 128.85, 128.61, 128.53, 128.43, 128.32, 116.22, 115.77, 80.40, 71.47, 70.86, 62.56;HRMS (ESI): m/z calcd. C_39_H_22_NaO_9_ [M + Na]^+^: 665.1788, found 665.1795.
