# Supplementary material for: Cyclohexene oxide CA, a derivative of zeylenone, exhibits anti-cancer activity in glioblastoma by inducing G0/G1 phase arrest through interference with EZH2
Source: Front Pharmacol. 2024 Jan 9;14:1326245. doi: 10.3389/fphar.2023.1326245 (PMC10803536; doi:10.3389/fphar.2023.1326245)
Supplement: Supplementary file 1 [file Presentation1.ZIP › Additional File 2.docx]

Table 1. The sh-RNA Sequence used in this study.

| Targets | | Sequences |
| --- | --- | --- |
| Sh-EZH2-1 | Sense | 5’-GAUAGAGAAUGUGGGUUUAdTdT-3’ |
|  | Anti-sense | 5’-UAAACCCACAUUCUCUAUCdTdT-3’ |
| Sh-EZH2-2 | Sense | 5’-GAAUGCCCUUGGUCAAUAUdTdT-3’ |
|  | Anti-sense | 5’-AUAUUGACCAAGGGCAUUCdTdT-3’ |
| Sh-NC | Sense | 5’-UUCUCCGAACGUGUCACGUdTdT-3’ |
|  | Anti-sense | 5’-ACGUGACACGUUCGGAGAAdTdT-3’ |

One-Step SYBR PrimeScript RT-PCR cycling conditions were as follows: 5 minutes at 42°C, 10 seconds at 95°C, 40 cycles of 3 seconds at 95°C, and 30 seconds at 60°C.

The reverse transcription was set as follows: 30 minutes at 16°C, 30 minutes at 42°C, and 5 minutes at 85°C. PCR conditions were set as follows: 2 minutes at 50°C, 10 minutes at 95°C, 40 cycles of 15 seconds at 95°C and 1 minutes at 60°C. GAPDH was used as the endogenous control. The relative expression levels were quantified as folder changes using the 2^-△△Ct^ methods.

Table 2. The PCR primers used in this study.

| Primers | | Sequences (5'->3') |
| --- | --- | --- |
| CDKN1B/p27 | Forward | 5’-GGGCAAGTACGAGTGGCAAGAG-3’ |
|  | Reverse | 5’-CAAATGCGTGTCCTCAGAGTTAGC-3’ |
| CDKN2A/p16 | Forward | 5’-CCCCGATTGAAAGAACCAGAGAG-3’ |
|  | Reverse | 5’-TACGGTAGTGGGGGAAGGCATA-3’ |
| GAPDH | Forward | 5’-CCCACTCCTCCACCTTTGAC-3’ |
|  | Reverse | 5’-CATACCAGGAAATGAGCTTGACAA-3’ |

Table 3. The qChIP-PCR primers that used in this study.

| Primers | | Sequences (5'->3') |
| --- | --- | --- |
| CDKN1B/p27 | Forward | 5’- ACTGTGCTTGGGAAGGAAGATCC-3’ |
|  | Reverse | 5’- CAGTGGGCAATGGTTCGCTC-3’ |
| CDKN2A/p16 | Forward | 5’-CCCCGATTCAATTTGGCAGTTAGG-3’ |
|  | Reverse | 5’- CAGCGTTGGCAAGGAAGGAGGAC-3’ |
