# Supplementary figures and images for: Cyclohexene oxide CA, a derivative of zeylenone, exhibits anti-cancer activity in glioblastoma by inducing G0/G1 phase arrest through interference with EZH2
Source: Front Pharmacol. 2024 Jan 9;14:1326245. doi: 10.3389/fphar.2023.1326245 (PMC10803536; doi:10.3389/fphar.2023.1326245)

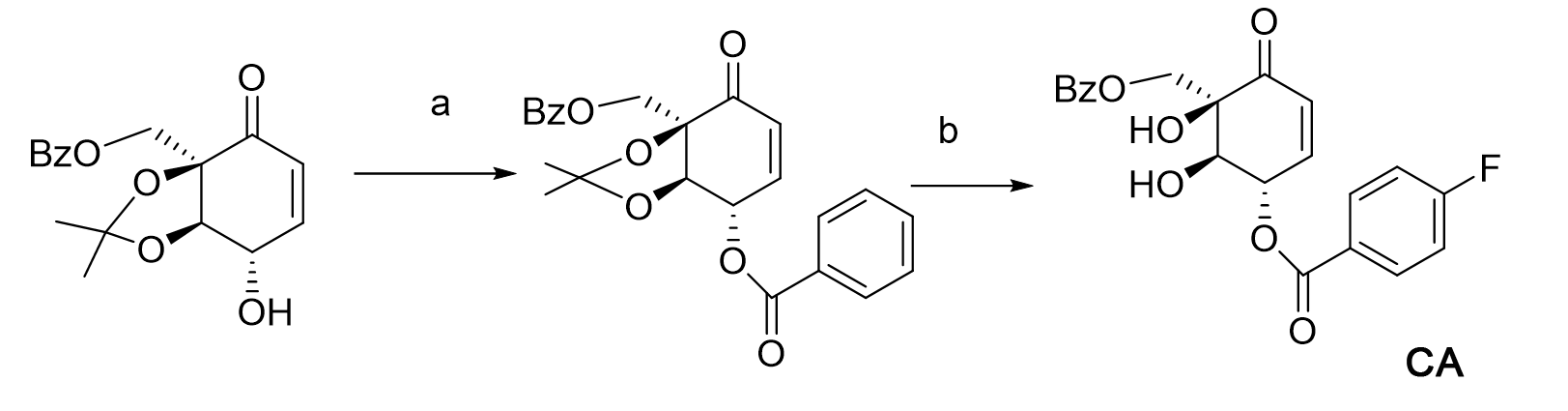

Supplement: Supplementary file 2 [file Image1.TIF]
